# Increasing rates of surgical treatment and preventing comorbidities may increase breast cancer survival for Aboriginal women

**DOI:** 10.1186/1471-2407-14-163

**Published:** 2014-03-07

**Authors:** Rajah Supramaniam, Alison Gibberd, Anthony Dillon, David Eamon Goldsbury, Dianne L O’Connell

**Affiliations:** 1 Cancer Research Division, Cancer Council NSW, Sydney, Australia; 2 School of Public Health, The University of Sydney, Sydney, Australia; 3 Institute for Positive Psychology and Education, Australia Catholic University, Sydney, Australia; 4 School of Public Health and Community Medicine, University of New South Wales, Sydney, Australia; 5 School of Medicine and Public Health, The University of Newcastle, Newcastle, Australia

**Keywords:** Australia/epidemiology, Breast Neoplasms/epidemiology, Female health services, Indigenous, Survival rate

## Abstract

**Background:**

Lower breast cancer survival has been reported for Australian Aboriginal women compared to non-Aboriginal women, however the reasons for this disparity have not been fully explored. We compared the surgical treatment and survival of Aboriginal and non-Aboriginal women diagnosed with breast cancer in New South Wales (NSW), Australia.

**Methods:**

We analysed NSW cancer registry records of breast cancers diagnosed in 2001–2007, linked to hospital inpatient episodes and deaths. We used unconditional logistic regression to compare the odds of Aboriginal and non-Aboriginal women receiving surgical treatment. Breast cancer-specific survival was examined using cumulative mortality curves and Cox proportional hazards regression models.

**Results:**

Of the 27 850 eligible women, 288 (1.03%) identified as Aboriginal. The Aboriginal women were younger and more likely to have advanced spread of disease when diagnosed than non-Aboriginal women. Aboriginal women were less likely than non-Aboriginal women to receive surgical treatment (odds ratio 0.59, 95% confidence interval (CI) 0.42-0.86). The five-year crude breast cancer-specific mortality was 6.1% higher for Aboriginal women (17.7%, 95% CI 12.9-23.2) compared with non-Aboriginal women (11.6%, 95% CI 11.2-12.0). After accounting for differences in age at diagnosis, year of diagnosis, spread of disease and surgical treatment received the risk of death from breast cancer was 39% higher in Aboriginal women (HR 1.39, 95% CI 1.01-1.86). Finally after also accounting for differences in comorbidities, socioeconomic disadvantage and place of residence the hazard ratio was reduced to 1.30 (95% CI 0.94-1.75).

**Conclusion:**

Preventing comorbidities and increasing rates of surgical treatment may increase breast cancer survival for NSW Aboriginal women.

## Background

Cancer contributes substantially to the difference in life expectancy between Aboriginal and non-Aboriginal women in Australia, and is the second highest cause of death in Aboriginal people [1,2]. Breast cancer is the most commonly diagnosed cancer in Aboriginal women, accounting for 25% of all incident cases in women [3].

It is generally reported that while breast cancer incidence in Aboriginal women is lower than in non-Aboriginal women [4-8], their population mortality rate from the disease is either lower [9] or similar, suggesting that the survival for Aboriginal women with breast cancer is lower [5,7,8,10]. This has been shown in the Northern Territory where breast cancer survival has been reported to be lower for Aboriginal women than for non-Aboriginal women [4]. The causes of breast cancer survival disparities for Aboriginal people are complex and have not been fully explained. The factors underlying these disparities are likely to include age at diagnosis, spread of disease at diagnosis, comorbidities, medical treatment received, socioeconomic disadvantage and access to health care. Studies in other Australian states have reported that compared to non-Aboriginal women, Aboriginal women diagnosed with breast cancer were more likely to have advanced spread of disease at diagnosis, multiple comorbidities, live in areas of higher socioeconomic disadvantage, and to live in areas further from major cancer treatment centres [2]. Most of the published research has focused on Aboriginal people living in sparsely populated, remote areas.

New South Wales (NSW) is the most populous state in Australia, with over 6.8 million residents [11]. It also has the largest number of Aboriginal residents (148 178), representing approximately 29% of the total Australian Aboriginal population [11]. Aboriginal people in NSW are more likely to live in metropolitan areas than those in other Australian states with Aboriginal populations of over 50,000 people. Nonetheless, Aboriginal people in NSW are still less likely to live in metropolitan areas (43%) than non-Aboriginal people (73%) [11].

As endorsed by the Aboriginal Health and Medical Research Council in NSW and in accordance with the NSW Health 2004 publication “Communicating Positively” we use the descriptor ‘Aboriginal people’ throughout this report to refer to the original people of Australia and their descendants [12].

Using population-based linked health records, we have compared the surgical treatment and survival of Aboriginal and non-Aboriginal women diagnosed with breast cancer in NSW. In particular, we investigated how the potentially modifiable factors of health care access and comorbidities influenced women’s treatment and survival.

## Methods

This analysis was conducted as part of the Aboriginal Patterns of Cancer Care Project (APOCC) which was funded by a National Health and Medical Research Council Health Services grant (Application Ref: 440202). This analysis was approved by the NSW Population and Health Service Research Ethics Committee and the Aboriginal Health and Medical Research Council Human Research Ethics Committee.

### Data sources

We analysed linked routinely collected population-based datasets of incident cancer cases, hospital inpatient episodes and deaths for NSW.

All invasive cancers diagnosed in NSW have been required to be notified to the NSW Central Cancer Registry (CCR) since 1972. We obtained data from the CCR for all invasive breast cancers (ICD-O-3 topography code C50 and morphology codes with a suffix of 3) diagnosed in 2001 to 2007 in women aged 18 years and over.

All inpatient episodes in public and private hospitals in NSW for these women were obtained from the NSW Ministry of Health’s Admitted Patient Data Collection (APDC) for the period 1 July 2000 to 30 June 2009.

Information on their vital status to 31 December 2008 was obtained from the NSW Registry of Births, Deaths and Marriages (RBDM). Deaths from breast cancer up to 31 December 2007 were obtained from the Australian Bureau of Statistics (ABS) and up to 31 December 2008 from the CCR.

The probabilistic linkage of the CCR, APDC, RBDM and ABS data was carried out by the Centre for Health Record Linkage (CHeReL) using ChoiceMaker software (ChoiceMaker Technologies Inc., New York, US). The CHeReL reviews all uncertain and samples of “certain” matches and non-matches of records, and reports approximately 0.1% false positive and less than 0.1% false negative linkages.

### Variables for analysis

It is mandatory to ask about Aboriginal status in all NSW public health facilities at each episode of care and Aboriginal status is a mandatory field for all NSW health data collection systems. In this analysis a woman was determined to be Aboriginal if she had identified that she was Aboriginal on a linked hospital admission or that she was identified as Aboriginal on her death certificate.

Women’s demographic and disease information obtained from the CCR included month and year of diagnosis, age and spread of disease at diagnosis. Spread of disease at diagnosis was reported by the CCR in four categories: localised, regional, distant and unknown.

Each woman was assigned to one of three categories according to the value of the Accessibility/Remoteness Index for Australia (ARIA+) [13] for her Local Government Area (LGA) of residence at the time of her diagnosis: major cities, inner regional or rural. The rural category included women living in outer regional, remote and very remote LGAs. The ARIA+ index is calculated using road distances of a LGA to the nearest population centres or ‘service centres’. The service centres are categorised into major cities, inner regional, outer regional, remote and very remote based on population size. The road distances for the LGA to the nearest service centre in each of the five categories is then divided by the Australian mean to create the LGA’s ARIA+ value [13]. Socioeconomic disadvantage was assigned to each woman according to the value of the ABS Socio-Economic Indexes for Areas Index of Relative Socio-Economic Advantage and Disadvantage (IRSAD) [14,15] for her LGA of residence at diagnosis. The IRSAD is a summary of census information about people and households within an area, including measures of income, education, types and sizes of housing and occupation [14,15]. LGAs were categorised into quintiles of socioeconomic disadvantage, with each quintile containing equal proportions of the NSW population.

Comorbidity information was derived from the APDC diagnosis codes, which recorded the reasons for admission and other conditions that may affect treatment or length of hospital stay. For each woman we noted any non-cancer condition described in the Charlson Comorbidity Index [16] in the 12 months prior to diagnosis and up to 6 months following breast cancer diagnosis in any hospital admission, including episodes where cancer was not the main reason for admission. The comorbidities were then grouped as the presence or absence of: diabetes, cardiovascular disease, chronic pulmonary disease (CPD) and any other non-cancer conditions. We excluded cancer as a comorbidity as we could not be certain that the cancer was independent of the current breast cancer diagnosis. This exclusion may have resulted in an underestimate of the overall impact of comorbidities on breast cancer mortality.

Breast cancer surgical treatments were identified in the APDC records by their ICD-10-AM codes and are reported here as the most radical treatment of either mastectomy (which may include a previous local excision/lumpectomy), local excision/lumpectomy only or no surgical treatment. We excluded 472 episodes of care that occurred more than two months prior to diagnosis as they may have been related to another primary breast cancer.

We restricted our analysis to surgical treatment, as other patterns of care studies have shown that there is high concordance between the surgical procedure recorded in the APDC and clinical audits of medical records, as surgical treatments invariably require the woman to be admitted pre- and/or post-operatively [17,18]. Conversely, adjuvant chemotherapy and radiotherapy treatments received were not assessed because they are largely administered as outpatient services and are therefore rarely recorded in the APDC.

### Statistical analysis

We used chi-squared tests to compare categorical patient characteristics between Aboriginal and non-Aboriginal women. The median number of days between diagnosis and surgery was compared using the non-parametric two-sided Wilcoxon rank sum test.

For women who had at least one linked APDC record in the time period between the 12 months prior and 6 months after their breast cancer diagnosis unconditional logistic regression was used to compare the odds of Aboriginal and non-Aboriginal women receiving surgical treatment. Variables were entered into the model using the method described in Hill et al. [19]. This involved first sequentially adjusting for factors relating to the woman (age at diagnosis and year of diagnosis) then the disease (spread of disease). Next, the potentially modifiable effects of factors relating to health care access (place of residence and socioeconomic disadvantage) and comorbidities were added to the model in order of their influence on the odds ratio for Aboriginal compared with non-Aboriginal women receiving surgical treatment.

Relative survival could not be estimated, as official lifetables are not available for NSW Aboriginal people. We therefore analysed breast cancer specific survival. Cumulative mortality curves [20] and Cox proportional hazards regression models were used to analyse the time to death from breast cancer after diagnosis and to adjust for known confounders respectively. The follow-up time for all women whose deaths were not recorded in any of the linked datasets was censored at 31 December 2008. For the Cox models, women who died from causes other than breast cancer were censored at the date of death. Variables were entered into the model using the method described above. As with the logistic regression we sequentially adjusted for the same factors relating to the woman and the disease, then for the potentially modifiable effects of comorbidities, surgical treatment, place of residence and socioeconomic disadvantage in order of their influence on the hazard ratio for breast cancer death for Aboriginal compared with non-Aboriginal women.

We tested for any significant interactions (p<0.05) between the variable indicating if a woman was Aboriginal and all other covariates in both the logistic regression and Cox proportional hazards regression. We also tested whether the proportional hazards assumption was satisfied by the final Cox regression model [21].

All statistical analyses were performed using SAS software (release 9.3; SAS Institute Inc, Cary, North Carolina) and R 2.15.1 [22].

## Results

There were 28 819 women diagnosed with primary breast cancer in NSW in the period 2001–2007. We excluded from the analysis 180 women (0.6% of all cases) who were diagnosed by death certificate or autopsy only. A further 789 women (2.8% of 28 639 remaining cases) who had no matching record in the APDC were also excluded; they are likely to be women who were treated in a neighbouring state [17].

Of the 27 850 remaining women, 288 (1.0%) identified as Aboriginal. Compared with non-Aboriginal women, Aboriginal women were diagnosed at younger age (median=57, interquartile range (IQR) 47–66) than non-Aboriginal women (median=59, IQR 50–70). Aboriginal women were also more likely to have regional or distant spread of disease, to live in rural or socioeconomically disadvantaged areas, and to have diabetes or chronic pulmonary disease (Table 1).

**Table 1:** Comparison of Aboriginal and non-Aboriginal women diagnosed with breast cancer in New South Wales 2001-2007

	**Aboriginal**		**Non-Aboriginal**		**p-value**
	**n**	**%**	**n**	**%**	
All women	288	1	27562	99	
Age at diagnosis (years)					<0.01
20-49	89	31	6621	24	
50-59	75	26	7209	26	
60-69	66	23	6446	23	
70-79	45	16	4426	16	
80+	13	5	2860	10	
Place of residence					<0.01
Major cities	138	48	20021	73	
Inner regional	90	31	5882	21	
Rural^a^	60	21	1659	6	
Spread of disease					0.04
Localised	133	46	14374	52	
Regional	112	39	9652	35	
Distant	24	8	1470	5	
Unknown	19	7	2066	7	
Socioeconomic disadvantage					<0.01
Least disadvantaged	23	8	5968	22	
Second least disadvantaged	42	15	6061	22	
Third least disadvantaged	35	12	4536	16	
Second most disadvantaged	74	26	5515	20	
Most disadvantaged	114	40	5482	20	
Breast cancer surgical treatment within 12 months of diagnosis					<0.01
No surgical treatment	43	15	3061	11	
Local excision/Lumpectomy only	106	37	13650	49	
Mastectomy^b^	139	48	10851	39	
Median (IQR^c^) days between diagnosis	15 (4–28)		14 (4–26)		0.27
And first breast cancer surgery					
Comorbidities^d^		n = 279		N = 26483	
Diabetes	49	18	2079	8	<0.01
Cardiovascular disease	20	7	1130	4	0.02
Chronic pulmonary disease	29	10	1012	4	<0.01
Other comorbidities	13	5	1093	4	0.66
No comorbidities recorded	195	70	22317	84	<0.01

^a^Includes outer regional, remote and very remote areas.

^b^Includes mastectomy with or without local excision/lumpectomy.

^c^IQR – interquartile range (25th and 75th centile).

^d^Numbers were reduced due to absence of linked hospital records during 12 months before and up to 6 months after breast cancer diagnosis. For Aboriginal women 9 did not have a linked record and 1161 non-Aboriginal women did not have a linked record in the time period. Note also that comorbidity categories are not mutually exclusive, so the percentages add to more than 100%.

Aboriginal women were less likely to receive surgical treatment than non-Aboriginal women (Table 1). More Aboriginal women had a mastectomy as their first surgery (46%) compared with non-Aboriginal women (34%). One year after diagnosis almost half of the Aboriginal women (48%) had undergone a mastectomy, compared with 39% of the non-Aboriginal women. For Aboriginal women living in major cities, inner regional and rural areas, within 12 months of diagnosis, 45%, 51% and 52% respectively had had a mastectomy compared with 39%, 42% and 42% respectively for non-Aboriginal women. The median time between diagnosis and the first surgical treatment was similar for Aboriginal (15 days) and non-Aboriginal women (14 days).

From our analysis of women who had at least one linked APDC record in the time period between the 12 months prior and 6 months after their breast cancer diagnosis, 87% of Aboriginal women compared with 92% of non-Aboriginal women received surgical treatment and the unadjusted odds ratio (OR) was 0.59 (95% Confidence Interval (CI) 0.42-0.86, p=0.006) (Table 2). After accounting for differences in age at diagnosis, year of diagnosis and spread of disease, Aboriginal women still had lower odds of receiving surgical treatment than non-Aboriginal women (OR 0.50, 95% CI 0.33-0.78, p=0.003). Finally, after accounting for comorbidities, place of residence at diagnosis and socioeconomic disadvantage the odds ratio for Aboriginal women receiving surgical treatment increased to be almost identical to the unadjusted value (OR 0.60, 95% CI 0.39-0.95, p=0.031). There were no significant interactions between the variable indicating if a woman identified as Aboriginal and any of the covariates described.

**Table 2:** Odds ratios for 279 Aboriginal women having breast cancer surgical treatment compared with 26483 non-Aboriginal women

**Covariate(s) adjusted for**^**a**^	**Odds ratio**^**b**^	**95% Confidence interval**	**p-value**
Aboriginal	0.59	0.42-0.86	0.006
+ Age at diagnosis	0.48	0.34-0.70	<0.001
+ Year of diagnosis	0.48	0.34-0.70	<0.001
+ Spread of disease	0.50	0.33-0.78	0.003
+ Place of residence	0.55	0.36-0.86	0.009
+ Comorbidities^c^	0.60	0.39-0.95	0.030
+ Socioeconomic disadvantage	0.60	0.39-0.95	0.031

^a^Covariates are entered sequentially and categories for each variable are as shown in Table 1. All subsequent models include the covariates from the previous model.

^b^For Aboriginal women compared with non-Aboriginal women.

^c^Comorbidities includes the presence or absence of: diabetes, cardiovascular disease, chronic pulmonary disease and any other (non-cancer) comorbidity in the Charlson Comorbidity Index.

All the factors listed in Table 3 were significantly associated with the risk of death from breast cancer for NSW women. However, in the multivariable model that included all factors shown in Table 3, diabetes, CPD, and place of residence were no longer significantly associated with the increased risk of death from breast cancer for NSW women. The differences in risk of death for Aboriginal and non-Aboriginal women was also no longer statistically significant after adjusting for all the factors in Table 3.

**Table 3:** Cox regression models of factors associated with breast cancer survival for NSW women 2001–2007 (n = 26762)

	**Unadjusted model**			**Multivariable model**		
	**Hazard ratio (95% CI)**		**p-value**	**Hazard ratio (95% CI)**		**p-value**
Aboriginal	1.69	1.22-2.25	0.002	1.30	0.94-1.75	0.105
Age at diagnosis			<0.001			<0.001
20-49	1.16	1.03-1.31		1.03	0.91-1.16	
50-69	1.00			1.00		
60-69	0.91	0.80-1.04		0.90	0.79-1.02	
70-79	1.71	1.51-1.93		1.52	1.35-1.72	
80+	3.52	3.12-3.97		2.27	1.99-2.59	
Year of diagnosis	0.97	0.95-1.00	0.018	0.96	0.94-0.98	<0.001
Spread of disease			<0.001			<0.001
Localised	1.00			1.00		
Regional	3.90	3.50-4.35		3.63	3.25-4.06	
Distant	24.46	21.76-27.53		11.85	10.40-13.50	
Unknown	5.40	4.64-6.29		2.41	2.05-2.83	
Comorbidities^a^						
Diabetes	1.52	1.33-1.72	<0.001	1.09	0.96-1.24	0.187
Cardiovascular disease	3.27	2.86-3.72	<0.001	1.18	1.02-1.37	0.030
Chronic pulmonary disease	1.63	1.37-1.92	<0.001	1.13	0.95-1.34	0.166
Other comorbidities	3.95	3.47-4.48	<0.001	1.60	1.38-1.84	<0.001
Surgical treatment			<0.001			<0.001
No surgical treatment	1.00			1.00		
Local excision/Lumpectomy only	0.06	0.06-0.07		0.17	0.15-0.19	
Mastectomy^b^	0.17	0.15-0.18		0.31	0.28-0.34	
Socioeconomic disadvantage			<0.001			0.018
Least disadvantaged	1.00			1.00		
Second least disadvantaged	1.14	1.00-1.29		1.09	0.96-1.24	
Third least disadvantaged	1.31	1.15-1.49		1.17	1.03-1.34	
Second most disadvantaged	1.33	1.17-1.50		1.25	1.09-1.42	
Most disadvantaged	1.35	1.19-1.52		1.19	1.02-1.38	
Place of residence			0.014			0.703
Major cities	1.00			1.00		
Inner regional	1.11	1.01-1.22		1.03	0.92-1.15	
Rural^b^	1.20	1.02-1.40		0.96	0.80-1.15	

^a^Presence compared with the absence for each comorbidity.

^b^Includes mastectomy with or without local excision/lumpectomy.

**Figure 1:**
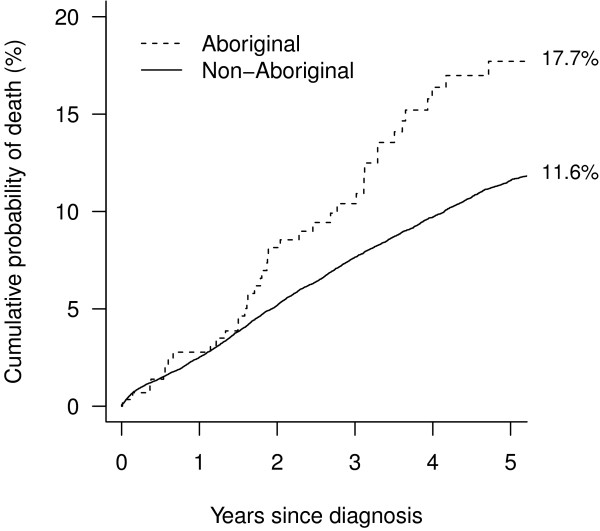
Cumulative mortality from breast cancer for 288 Aboriginal and 27562 non-Aboriginal women diagnosed in NSW, 2001–2007.

The five-year crude breast cancer-specific mortality was 6.1% higher for Aboriginal women (17.7%, 95% CI: 12.9-23.2) compared with non-Aboriginal women (11.6%, 95% CI: 11.2-12.0) (Figure 1). Aboriginal women had a 69% higher unadjusted risk of breast cancer death relative to non-Aboriginal women (Hazard ratio (HR) 1.69, 95% CI 1.22-2.25, p=0.002) (Table 4). The hazard ratio for Aboriginal women compared with non-Aboriginal women was similar to the unadjusted value at 1.67 (95% CI 1.21-2.23, p=0.002) after adjusting for differences in age at diagnosis, year of diagnosis and spread of disease (Table 4). However after adjusting for surgical treatment the hazard ratio decreased to 1.39 (95% CI 1.01-1.86, p=0.045). Also, after accounting for comorbidities the risk of death from breast cancer for Aboriginal women was still 34% higher than for non-Aboriginal women, however this difference was not statistically significant (HR 1.34, 95% CI 0.97-1.79, p=0.075). Finally, after also accounting for differences in socioeconomic disadvantage and place of residence, there was little change in the hazard ratio (HR 1.30, 95% CI 0.94-1.75, p=0.105). This final model satisfied the proportional hazards assumption.

**Table 4:** Hazard ratios for 279 Aboriginal women dying from breast cancer compared with 26483 non-Aboriginal women

**Covariate(s) adjusted for**^**a**^	**Hazard ratio**^**b**^	**95% Confidence interval**	**p-value**
Aboriginal	1.69	1.22-2.25	0.002
+ Age at diagnosis	1.88	1.36-2.51	<0.001
+ Year of diagnosis	1.88	1.36-2.51	<0.001
+ Spread of disease	1.67	1.21-2.23	0.002
+ Surgery	1.39	1.01-1.86	0.045
+ Comorbidities^c^	1.34	0.97-1.79	0.075
+ Socioeconomic disadvantage	1.30	0.94-1.74	0.109
+ Place of residence	1.30	0.94-1.75	0.105

^a^Covariates are entered sequentially and categories for each variable are as shown in Table 3. All subsequent models include the covariates from the previous model.

^b^For Aboriginal women compared with non-Aboriginal women.

^c^Comorbidities include the presence of absence of: diabetes, cardiovascular disease, chronic pulmonary disease and any other (non-cancer) comorbidity in the Charlson Comorbidity Index.

## Discussion

We found that in NSW, Australia, Aboriginal women diagnosed with breast cancer were significantly less likely to receive surgical treatment and had poorer survival than non-Aboriginal women. The disparity in surgical treatment was not accounted for by differences in age at diagnosis, year of diagnosis, spread of disease, comorbidities, place of residence at diagnosis or socioeconomic disadvantage. After accounting for differences in age at diagnosis, year of diagnosis and spread of disease Aboriginal women had a 67% higher risk of death from breast cancer than non-Aboriginal women. However this increased risk was reduced after accounting for differences in the potentially modifiable factors of surgical treatment received and comorbidities. These results suggest that increasing rates of surgical treatment and preventing comorbidities may increase the survival of Aboriginal women diagnosed with breast cancer in NSW.

Others have identified that place of residence and socioeconomic disadvantage manifest as barriers to treatment uptake through lack of transportation, accommodation and/or childcare facilities [23]. Our finding that Aboriginal women were more likely to have a mastectomy, also found in previous studies [24,25], may be a result not only of more advanced disease at diagnosis, but also of poorer access to adjuvant therapies. The proportion having mastectomy increased with increasing remoteness of residence indicating that this may be the case. However women may have also chosen a mastectomy over a lumpectomy to reduce the number of visits required for treatment. It is also possible that having significant comorbidities limits women’s access to surgery and adjuvant therapies, as they may have lower tolerance of, or ability to recover from, these treatments [26]. If the prevalence of comorbidities was reduced and access to, and acceptability of, health care services improved, the disparities in surgical treatment rates and survival from breast cancer between NSW Aboriginal and non-Aboriginal women may be reduced.

Free mammographic screening has been available through BreastScreen NSW since 1991. In the period 2000–2008 participation by Aboriginal women in the program ranged between 30% and 35% compared to between 50% and 56% for non-Aboriginal women [27]. This difference in participation may explain some of the differences in stage at diagnosis between Aboriginal and non-Aboriginal women. It is however, unlikely to be the only factor, as more than half of all breast cancers are diagnosed outside BreastScreen NSW’s target age group of 50–69, and only 40% of cancers diagnosed annually in the target age group are diagnosed through the screening program [27].

A limitation of this study is the identification of Aboriginal women in our source datasets, although this was addressed by using any record of a woman identifying as Aboriginal by the ABS death records or in any hospital admission record, including non-cancer related admissions, to indicate that a woman was Aboriginal. As 98% of the NSW population is non-Aboriginal the chance of a false positive misclassification is low. The NSW Ministry of Health has recently evaluated a number of different algorithms for improving identification of Aboriginal people in linked health data sets and found that an ‘ever identified’ algorithm, as used here, increased the identification of Aboriginal people in the CCR by 49% [28]. Finally, although recent efforts to improve the identification of Aboriginal people in health data systems have been largely successful [29], it is likely that any statistics presented here still underestimate the number of Aboriginal women diagnosed with breast cancer in NSW. A recent paper describing the same population, but for a different time period, estimated the under-reporting of Aboriginal identification to be between 12% and 14% [30]. If the unidentified Aboriginal women had better outcomes than those who were identified, the results of this study would likely be biased away from the null hypotheses of no differences in surgical treatment or survival rates for Aboriginal and non-Aboriginal women. A further limitation is the potential under-reporting of comorbidities in the hospital records, although diabetes has been shown to be reasonably reliably recorded [18]. Another limitation is that we were unable to measure other reasons besides access factors that may contribute to the observed lower surgical treatment rates for Aboriginal women. A qualitative study in NSW identified several cultural barriers between Aboriginal people and mainstream cancer services that may be subtly contributing to lower usage or acceptability of some cancer treatments. In particular it identified lower cancer literacy for Aboriginal people [31], a feeling of a lack of social inclusion when in hospital settings [32], and the need for health services to openly discuss and address cultural differences in service delivery [33].

A final potential limitation is a possible bias in our survival comparisons due to the identification of Aboriginal women through the ABS death records. We investigated the magnitude of this effect in a sensitivity analysis where we determined Aboriginal status from the APDC only. There were nine women who were identified as Aboriginal by their ABS records only (of whom five died of breast cancer and four died of other causes and were censored in our analysis). When these nine women were excluded from the analysis, the results and conclusions were unchanged as the fully adjusted hazard ratio comparing survival in Aboriginal and non-Aboriginal women decreased slightly from 1.30 (95% CI 0.94-1.75) to 1.19 (95% CI: 0.84-1.63).

The strengths of our study include the whole population approach to identifying cases. This is the first such study conducted in NSW, the Australian state with the largest Aboriginal population, including the largest number of Aboriginal women living in metropolitan areas. This is also the first study to include detailed information on comorbidities and to assess their effects on surgical treatment and survival for Aboriginal women. Our study is also the first to statistically account for the disparities in the risk of breast cancer specific death by adjusting for differences in comorbidities and surgical treatment received in addition to age at diagnosis, year of diagnosis, spread of disease, place of residence and socioeconomic disadvantage. Previous published studies on breast cancer survival for Aboriginal women have only adjusted for differences in some of these covariates and so were unable to discern the relative or combined effects of all of the covariates [10,34].

Our results for NSW Aboriginal women, compared to non-Aboriginal women, broadly concurred with the lower incidence of breast cancer yet similar mortality rate for the whole population observed in the less populous jurisdictions of Australia [4,5,7] suggesting poorer survival for Aboriginal women. We found that Aboriginal women in NSW were 69% more likely to die from their breast cancer than non-Aboriginal women, while studies from the Northern Territory and South Australia found that Aboriginal women were almost twice as likely as non-Aboriginal women to die of their breast cancer [4,34]. The Northern Territory study also found that differences in age and stage did not explain the survival gap between Aboriginal and non-Aboriginal women [4]. Similar disparities in the rates of surgical treatment between Aboriginal and non-Aboriginal women to those we have reported were found in Queensland and Western Australia [35,36].

Our results also seem comparable to international studies of cancer in Indigenous populations. A recent study in New Zealand found that Maori women had lower breast cancer survival than non-Maori women, and that this difference could not be accounted for by disparities in age and spread of disease [37]. Studies of women with breast cancer in the United States have also found poorer survival for American Indian, Alaskan Native and Hawaiian Native women compared to their respective non-Indigenous populations [38-40], and again, differences in age and spread of disease did not explain this disparity. Studies found that the surgical treatment rates for American Indian and Alaskan Native women with breast cancer were similar to those for non-Indigenous women, however the time between diagnosis and treatment was longer [41,42].

## Conclusions

Aboriginal women in NSW diagnosed with breast cancer received less surgical treatment than non-Aboriginal women. Aboriginal women were also less likely to survive their breast cancer than non-Aboriginal women. We have shown that the disparity in survival could be reduced by preventing comorbidities and increasing rates of surgical treatment.

## Competing interests

The authors declare that they have no competing interests.

## Authors’ contributions

All authors listed in this paper fulfil the criteria of authorship, and there is no one else who fulfils these criteria who is not listed here as an author. Contributions were as follows: RS contributed to study design, led data analysis and interpretation, and wrote all drafts of the paper; AG conducted the data analysis and contributed to the interpretation and to draft revisions; DG contributed to data analysis, interpretation and to draft revisions; AD contributed to interpretation and to draft revisions; DO’C contributed to study design, data interpretation and draft revisions. All authors read and approved the final manuscript.

## Pre-publication history

The pre-publication history for this paper can be accessed here:

http://www.biomedcentral.com/1471-2407/14/163/prepub

## References

[B1] Zhao Y Dempsey K Causes of inequality in life expectancy between Indigenous and non-Indigenous people in the Northern Territory, 1981–2000: a decomposition analysis Med J Aust 2006 184 490 494 16719745 10.5694/j.1326-5377.2006.tb00340.x

[B2] Australian Institute of Health and Welfare The Health and Welfare of Australia’s Aboriginal and Torres Strait Islander People an Overview 2011 Canberra: AIHW

[B3] Pink B Allbon P The Health and Welfare of Australia’s Aboriginal and Torres Strait Islander Peoples 2008 Canberra: Australian Institute of Health and Welfare

[B4] Condon JR Hock L Garling LS Cancer survival Northern territory 1991–2001 2006 Department of Health and Community Services, Darwin: Northern Territory Cancer Registry

[B5] Moore SP O’Rourke PK Mallitt K-A Garvey G Green AC Coory MD Valery PC Cancer incidence and mortality in Indigenous Australians in Queensland, 1997–2006 Med J Aust 2010 193 590 593 21077815 10.5694/j.1326-5377.2010.tb04068.x

[B6] Cunningham J Rumbold AR Zhang X Condon JR Incidence, aetiology, and outcomes of cancer in Indigenous peoples in Australia Lancet Oncol 2008 9 585 595 10.1016/S1470-2045(08)70150-5 18510990

[B7] Australian Institute of Health and Welfare, Cancer Australia Breast Cancer in Australia: An Overview 2012 Canberra ACT, Australia: AIHW

[B8] Threlfall T Thompson J Cancer incidence and mortality in Western Australia, 2007 2009 Western Australia, Perth: Department of Health

[B9] Supramaniam R Grindley H Pulver LJ Cancer mortality in Aboriginal people in New South Wales, Australia, 1994–2002 Aust N Z J Public Health 2006 30 453 456 10.1111/j.1467-842X.2006.tb00463.x 17073228

[B10] Condon JR Barnes T Armstrong BK Selva-Nayagam S Elwood JM Stage at diagnosis and cancer survival for indigenous Australians in the Northern territory Med J Aust 2005 182 277 280 15777142 10.5694/j.1326-5377.2005.tb06700.x

[B11] Australian Bureau of Statistics Population characteristics, Aboriginal and Torres Strait Islander Australians 2010 Canberra: ABS

[B12] NSW Department of Health Communicating positively a guide to appropriate Aboriginal terminology. Volume 14 2004 Sydney: NSW Department of Health

[B13] Department of Health and Aged Care Measuring Remoteness: Accessibility/Remoteness Index of Australia (ARIA) 2001 Canberra: Department of Health and Aged Care

[B14] Australian Bureau of Statistics Census of Population and Housing: Socio-Economic Indexes for Areas (SEIFA), Australia - Data Cube only, 2001 2006 Canberra: ABS

[B15] Australian Bureau of Statistics Census of Population and Housing: Socio-Economic Indexes for Areas (SEIFA), Australia - Data Cube only, 2006. 2033.0.30 2008 Canberra: ABS

[B16] Charlson ME Pompei P Ales KL MacKenzie CR A new method of classifying prognostic comorbidity in longitudinal studies: development and validation J Chronic Dis 1987 40 373 383 10.1016/0021-9681(87)90171-8 3558716

[B17] Goldsbury DE Smith DP Armstrong BK O’Connell DL Using linked routinely collected health data to describe prostate cancer treatment in New South Wales, Australia: a validation study BMC Health Serv Res 2011 11 253 10.1186/1472-6963-11-253 21978077 PMC3206422

[B18] Goldsbury DE Armstrong K Simonella L Armstrong BK O’Connell DL Using administrative health data to describe colorectal and lung cancer care in New South Wales, Australia: a validation study BMC Health Serv Res 2012 12 387 10.1186/1472-6963-12-387 23140341 PMC3512511

[B19] Hill S Sarfati D Blakely T Robson B Purdie G Chen J Dennett E Cormack D Cunningham R Dew K McCreanor T Kawachi I Survival disparities in Indigenous and non-Indigenous New Zealanders with colon cancer: the role of patient comorbidity, treatment and health service factors J Epidemiol Community Health 2010 64 117 123 10.1136/jech.2008.083816 20056966

[B20] Gray RJ A class of K-sample tests for comparing the cumulative incidence of a competing risk Ann Stat 1988 16 1141 1154 10.1214/aos/1176350951

[B21] Grambsch PM Thernau TM Proportional hazards tests and diagnostics based on weighted residuals Biometrika 1994 81 515 526 10.1093/biomet/81.3.515

[B22] R Development Core Team R: A Language and Environment for Statistical Computing 2012 Vienna, Austria: R Foundation for Statistical Computing http://www.R-project.org/

[B23] Thompson SC Shahid S Bessarab D Durey A Davidson PM Not just bricks and mortar: planning hospital cancer services for Aboriginal people BMC Res Notes 2011 4 62 10.1186/1756-0500-4-62 21401923 PMC3068108

[B24] Heathcote K Armstrong BK Disparities in cancer outcomes in regional and rural Australia Cancer Forum 2007 31 70 74

[B25] Elder EE Hay SB Moore K Factors influencing treatment recommendations in node-negative breast cancer J Oncol Pract 2011 7 26 30 10.1200/JOP.2010.000024 21532807 PMC3014507

[B26] Stavrou EP Lu CY Buckley N Pearson S The role of comorbidities on the uptake of systemic treatment and 3-year survival in older cancer patients Ann Oncol 2012 23 2422 2428 10.1093/annonc/mdr618 22351742

[B27] Australian Institute of Health and Welfare BreastScreen Australia Monitoring Report 2006–2007 and 2007–2008 2010 Canberra: AIHW

[B28] Population and Public Health Division Improved reporting of Aboriginal and Torres Strait Islander Peoples on population datasets in New South Wales using record linkage – a feasibility study 2012 Sydney: NSW Ministry of Health

[B29] Australian Institute of Health and Welfare Indigenous Identification in Hospital Separations Data – Quality Report 2010 Canberra: AIHW

[B30] Morrell S You H Baker D Estimates of cancer incidence, mortality and survival in Aboriginal people from NSW, Australia BMC Cancer 2012 12 168 10.1186/1471-2407-12-168 22559220 PMC3520119

[B31] Treloar C Gray R Brener L Jackson C Saunders V Johnson P Harris M Butow P Newman C Health literacy in relation to cancer: addressing the silence about and absence of cancer discussion among Aboriginal people, communities and health services Health Soc Care Communit 2013 21 655 664 10.1111/hsc.12054 23692557

[B32] Treloar C Gray R Brener L Jackson C Saunders V Johnson P Harris M Butow P Newman C “I can’t do this, it’s too much”: building social inclusion in cancer diagnosis and treatment experiences of Aboriginal people, their carers and health workers 2013 Epub: Int J Public Health 10.1007/s00038-013-0466-1 23604078

[B33] Newman CE Gray R Brener L Jackson LC Johnson P Saunders V Harris M Butow P Treloar C One size fits all? The discursive framing of cultural difference in health professional accounts of providing cancer care to Aboriginal people Ethn Health 2013 18 433 447 10.1080/13557858.2012.754408 23297651

[B34] Chong A Roder D Exploring differences in survival from cancer among Indigenous and non-Indigenous Australians: implications for health service delivery and research Asian Pac J Cancer Prev 2010 11 953 961 21133607

[B35] Shaw IM Elston TJ Retrospective, 5-year surgical audit comparing breast cancer in indigenous and non-indigenous women in Far North Queensland ANZ J Surg 2003 73 758 760 10.1046/j.1445-2197.2003.02751.x 12956794

[B36] Hall SE Bulsara CE Bulsara MK Leahy TG Culbong MR Hendrie D Holman CDJ Treatment patterns for cancer in Western Australia: does being Indigenous make a difference? Med J Aust 2004 181 191 194 15310252 10.5694/j.1326-5377.2004.tb06234.x

[B37] Robson B Purdie G Cormack D Unequal impact: Māori and Non-Māori cancer statistics 1996–2001 2006 Wellington: New Zealand Ministry of Health

[B38] Harper S Lynch J Meersman SC Breen N Davis WW Reichman MC Trends in area-socioeconomic and race-ethnic disparities in breast cancer incidence, stage at diagnosis, screening, mortality, and survival among women ages 50 years and over (1987–2005) Cancer Epidemiol Biomarkers Prev 2009 18 121 131 10.1158/1055-9965.EPI-08-0679 19124489

[B39] Braun K Fong M Gotay C Pagano I Chong C Ethnicity and breast cancer in Hawaii: increased survival but continued disparity Ethn Dis 2005 15 453 460 16108306

[B40] Li CI Malone KE Daling JR Differences in breast cancer stage, treatment, and survival by race and ethnicity Arch Intern Med 2003 163 49 56 10.1001/archinte.163.1.49 12523916

[B41] Wilson RT Adams-Cameron M Burhansstipanov L Roubidoux MA Cobb N Lynch CF Edwards BK Disparities in breast cancer treatment among American Indian, Hispanic and non-Hispanic White Women Enrolled in Medicare J Health Care Poor Underserved 2007 18 648 664 10.1353/hpu.2007.0071 17675720

[B42] Day GE Kelly JJ Lanier AP Murphy N Women’s cancers among Alaska Natives 1969–2003 Alaska Med 2007 49 2 Suppl 91 94 17929614

